# Supplementary material for: Bifunctional architecture enables substrate catalysis and channeling in Paracoccus TMAO demethylase
Source: eLife. 2026 Sep 28;15:RP109964. doi: 10.7554/eLife.109964 (PMC13619132; doi:10.7554/eLife.109964)
Supplement: Peer review history

# Editor report: eLife Assessment

**DOI:** 10.7554/eLife.109964.4.sa0

**Authors:** Julien Roche

**Affiliations:** Iowa State University United States

**Keywords:** Important, Incomplete

This study reports high-resolution cryo-EM structures of Paracoccus trimethylamine N-oxide demethylase and advances the intriguing hypothesis that the enzyme may be bifunctional, coupling TMAO demethylation to formaldehyde capture at a distal tetrahydrofolate-binding site through an enclosed intramolecular tunnel. Supported by biochemical assays and molecular dynamics simulations, the structural findings are **important** and potentially of broad interest, particularly the unusual oligomeric architecture and the proposed conduit for a reactive intermediate. However, evidence that the conduit mediates formaldehyde channeling remains **incomplete**, because the current HCHO-THF measurements do not demonstrate enzyme-dependent transfer or distinguish downstream HCHO consumption from indirect effects of THF on enzyme activity.

# Referee report: Reviewer #1 (Public review)

**DOI:** 10.7554/eLife.109964.4.sa1

Summary:

Thach et al. report on the structure and function of trimethylamine N-oxide demethylase (TDM). They identify a novel complex assembly composed of multiple TDM monomers and obtain high-resolution structural information for the catalytic site, including an analysis of its metal composition, which leads them to propose a mechanism for the catalytic reaction.

In addition, the authors describe a novel substrate channel within the TDM complex that connects the N-terminal Zn²⁺-dependent TMAO demethylation domain with the C-terminal tetrahydrofolate (THF)-binding domain. This continuous intramolecular tunnel appears highly optimized for shuttling formaldehyde (HCHO), based on its negative electrostatic properties and restricted width. The authors propose the hypothesis that this channel facilitates the safe transfer of HCHO, enabling its efficient conversion to methylenetetrahydrofolate (MTHF) at the C-terminal domain as a microbial detoxification strategy. Experimental data that shows an involvement of TDM in the reaction of HCHO with THF is less convincing.

Strengths:

The authors provide convincing high-resolution cryo-EM structural evidence (up to 2 Å) revealing an intriguing complex composed of two full monomers and two half-domains. They further present evidence for the metal ion bound at the active site and articulate a hypothesis for the catalytic cycle. Substantial effort is devoted to optimizing and characterizing enzyme activity, including detailed kinetic analyses across a range of pH values, temperatures, and substrate concentrations. Furthermore, the authors validate their structural insights through functional analysis of active-site point mutants.

In addition, the authors identify a continuous channel for formaldehyde (HCHO) passage within the structure and support this interpretation through molecular dynamics simulations. These analyses suggest an exciting mechanism of specific, dynamic, and gated channeling of HCHO. This finding is particularly appealing, as it implies the existence of a unique, completely enclosed conduit that may be of broad interest, including potential applications in bioengineering.

Weaknesses:

Although the idea of an enclosed channel for HCHO is compelling, the experimental evidence supporting enzymatic assistance in the reaction of HCHO with THF is less convincing. The linear regression analysis shown in Figure 1C demonstrates a THF concentration-dependent decrease in HCHO; however, it is well established that HCHO and THF can react spontaneously in a non-enzymatic manner, raising the possibility that the observed effect does not require enzymatic involvement. I appreciate the authors' clarification that the data in Figure 1 were not intended to demonstrate enzymatic channeling or catalytic involvement in the HCHO-THF reaction, and that the assay does not distinguish between changes in HCHO production and downstream consumption. The authors' revised statement "Overall, these findings suggest that TDM-mediated TMAO demethylation generates HCHO, which can subsequently react with THF, potentially linking TMAO breakdown to one-carbon metabolism." is appropriately cautious and leaves open the mechanism underlying this process, which is consistent with the evidence presented.

Overall, the authors were successful in advancing our structural and functional understanding of the TDM complex. They suggest an interesting oligomeric complex composition which in my opinion should be investigated with additional biophysical techniques.

Additionally, they provide an intriguing hypothesis for a new type of substrate channeling. However, additional kinetic experiments focusing on HCHO and THF turnover by enzymatic proximity effects are required to strengthen this potentially fundamental finding. If this channeling mechanism can be supported by stronger experimental evidence, it would substantially advance our understanding and knowledge of biologic conduits and enable future efforts in the design of artificial cascade catalysis systems with high conversion rate and efficiency, as well as detoxification pathways.

Comments on revised version.

It is unfortunate that no additional experimental evidence supporting the proposed channeling mechanism could be provided. In the absence of such evidence, I remain somewhat hesitant to regard the evidence as "solid" rather than "incomplete," particularly given that the authors themselves acknowledge that the current experiments do not establish enzymatic channeling. However, the rest of the manuscript is stronger despite this limitation.

# Author comment: Author response

**DOI:** 10.7554/eLife.109964.4.sa2

**Authors:** Trung Thach, KanagaVijayan Dhanabalan, Shiwangi Maurya, Yu Han-Hallett, Senwei Quan, Jane Allison, Gurunath Ramanathan, Ramaswamy Subramanian

**Affiliations:** Purdue University West Lafayette West Lafayette United States; Purdue University West Lafayette West Lafayette United States; Indian Institute of Technology Kanpur Kanpur India; Purdue University West Lafayette West Lafayette United States; University of Auckland Auckland New Zealand; University of Auckland Auckland New Zealand; Indian Institute of Technology Kanpur Kanpur India; Purdue University West Lafayette West Lafayette United States

The following is the authors’ response to the previous reviews

**Public Reviews:**

**Reviewer #1 (Public review):**

Summary:

Thach et al. report on the structure and function of trimethylamine N-oxide demethylase (TDM). They identify a novel complex assembly composed of multiple TDM monomers and obtain high-resolution structural information for the catalytic site, including an analysis of its metal composition, which leads them to propose a mechanism for the catalytic reaction.

In addition, the authors describe a novel substrate channel within the TDM complex that connects the N-terminal Zn^2+^-dependent TMAO demethylation domain with the C-terminal tetrahydrofolate (THF)-binding domain. This continuous intramolecular tunnel appears highly optimized for shuttling formaldehyde (HCHO), based on its negative electrostatic properties and restricted width. The authors propose that this channel facilitates the safe transfer of HCHO, enabling its efficient conversion to methylenetetrahydrofolate (MTHF) at the C-terminal domain as a microbial detoxification strategy. Experimental data that shows an involvement of TDM in the reaction of HCHO with THF is less convincing.

Strengths:

The authors provide convincing high-resolution cryo-EM structural evidence (up to 2 Å) revealing an intriguing complex composed of two full monomers and two half-domains. They further present evidence for the metal ion bound at the active site and articulate a hypothesis for the catalytic cycle. Substantial effort is devoted to optimizing and characterizing enzyme activity, including detailed kinetic analyses across a range of pH values, temperatures, and substrate concentrations. Furthermore, the authors validate their structural insights through functional analysis of active-site point mutants.

In addition, the authors identify a continuous channel for formaldehyde (HCHO) passage within the structure and support this interpretation through molecular dynamics simulations. These analyses suggest an exciting mechanism of specific, dynamic, and gated channelling of HCHO. This finding is particularly appealing, as it implies the existence of a unique, completely enclosed conduit that may be of broad interest, including potential applications in bioengineering.

Weaknesses:

Although the idea of an enclosed channel for HCHO is compelling, the experimental evidence supporting enzymatic assistance in the reaction of HCHO with THF is less convincing. The linear regression analysis shown in Figure 1C demonstrates a THF concentration-dependent decrease in HCHO; however, it is well established that HCHO and THF can react spontaneously in a non-enzymatic manner, raising the possibility that the observed effect does not require enzymatic involvement. I appreciate the authors' clarification that the data in Figure 1 were not intended to demonstrate enzymatic channelling or catalytic involvement in the HCHO-THF reaction, and that the assay does not distinguish between changes in HCHO production and downstream consumption. However, the statement "these findings show that TDM carries out two linked reactions: TMAO demethylation at one active site, and the HCHO produced can condense with THF at the C-terminal domain, connecting TMAO breakdown to one-carbon metabolism" (page 2) still implies a mechanistic and functional coupling that is not supported by the presented data and appears inconsistent with the authors' clarification. In light of this, I recommend revising this statement to avoid implying mechanistic or functional coupling between the two reactions unless additional experimental evidence is provided.

We thank the reviewer for this clarification. We have revised as per recommendation (page 2).

“Overall, these findings suggest that TDM-mediated TMAO demethylation generates HCHO, which can subsequently react with THF, potentially linking TMAO breakdown to one-carbon metabolism.”

Overall, the authors were successful in advancing our structural and functional understanding of the TDM complex. They suggest an interesting oligomeric complex composition which should be investigated with additional biophysical techniques.

Additionally, they provide an intriguing hypothesis for a new type of substrate channelling. Additional kinetic experiments focusing on HCHO and THF turnover by enzymatic proximity effects would strengthen this potentially fundamental finding. If this channelling mechanism can be supported by stronger experimental evidence, it would substantially advance our understanding and knowledge of biologic conduits and enable future efforts in the design of artificial cascade catalysis systems with high conversion rate and efficiency, as well as detoxification pathways.

**Reviewer #2 (Public review):**

Summary:

The manuscript reports a cryo-EM structure of TMAO demethylase from Paracoccus sp. This is an important enzyme in the metabolism of trimethylamine oxide (TMAO) and trimethylamine (TMA) in human gut microbiota, so new information about this enzyme would certainly be of interest.

Strengths:

The cryo-EM structure for this enzyme is new and provides new insights into the function of the different protein domains, and a channel for formaldehyde between the two domains.

Weaknesses:

(1) The proposed catalytic mechanism in this manuscript does not make sense. Previous mechanistic studies on the Methylocella silvestris TMAO demethylase (FEBS Journal 2016, 283, 3979-3993, reference 7) reported that, as well as a Zn2+ cofactor, there was a dependence upon non-heme Fe2+, and proposed a catalytic mechanism involving deoxygenation to form TMA and an iron(IV)-oxo species, followed by oxidative demethylation to form DMA and formaldehyde.

In this work, the authors do not mention the previously proposed mechanism, but instead just say that elemental analysis "excluded iron". This is alarming, since the previous work has a key role for non-heme iron in the mechanism. The elemental analysis here gives a Zn content of about 0.5 mol/mol protein (and no Fe), whereas the Methylocella TMAO demethylase was reported to contain 0.97 mol Zn/mol protein, and 0.35-0.38 mol Fe/mol protein. It does, therefore, appear that their enzyme is depleted in Zn, and the absence of Fe impacts on the mechanism, as explained below.

The proposed catalytic mechanism in this manuscript, I am sorry to say, does not make sense, for several reasons:

(i) Demethylation to form formaldehyde is not a hydrolytic process; it is an oxidative process (normally accomplished by either cytochrome P450 or non-heme iron-dependent oxygenase). The authors propose that a zinc (II) hydroxide attacks the methyl group, which (a) is unprecedented, (b) even if it were possible, would generate methanol, not formaldehyde.

(ii) The amine oxide is proposed to deoxygenate, with hydroxide appearing on the Zn - unfortunately, amine oxide deoxygenation is a reductive process, for which a reducing agent is needed, and Zn2+ is not a redox-active metal ion;

(iii) The authors say "forming a tetrahedral intermediate, as described for metalloprotease," but zinc metalloproteases attack an amide carbonyl to form an oxyanion intermediate, whereas in this mechanism, there is no carbonyl to attack, so this statement is just wrong.

So on several counts the proposed mechanism cannot be correct. Some redox cofactor is needed in order to carry out amine oxide deoxygenation, and Zn2+ cannot fulfil that role. Fe2+ could do, which is why the previously proposed mechanism involving an iron(IV)-oxo intermediate is feasible. But the authors claim that their enzyme has no Fe. If so then there must be some other redox cofactor present. Therefore, the authors need to re-analyse their enzyme carefully and look either for Fe or for some other redox-active metal ion, and then provide convincing experimental evidence for a feasible catalytic mechanism. As it stands the proposed catalytic mechanism is unacceptable.

Revised version. The authors have essentially not changed the proposed mechanism. They have removed the reference to zinc metalloproteases, but still propose a mechanism mediated only by Zn2+. As explained above, attack by zinc (II) hydroxide is unprecedented and would generate methanol, not formaldehyde, and amine deoxygenation is a reductive process that cannot be fulfilled by Zn2+. So the proposed mechanism is still not feasible at all. The authors now say that "oxidative chemistry....remains unresolved", I'm sorry, but that is not acceptable.

I have urged the authors to re-examine the metal content of their enzyme, In the Supporting Information (Figure S5) they give ICPMS data that indicates a Zn stoichiometry of 0.5 mol Zn/mol protein, and Fe is not detected. Have the authors analysed for other redox active metals? The authors say that there is no evidence for any other metal binding site, but there is only 50% occupancy of Zn in their protein, so could there be a different metal ion present in place of Zn in the other 50% of the protein, that accounts for the observed activity?

Since there is clearly a major discrepancy here, the onus is on the authors to explain the discrepancy, rather than just returning with the same data. For example, they could treat the enzyme with EDTA to remove all metals (and check the treated enzyme by ICPMS), and then add different metal ions to test activity with different metals (could even titrate with different molar equivalents of metal ions). They could then test a range of different redox-active metal ions.

We have re-examined our data and repeated experiments on the reviewer's opinion. We have repeated the IC-PMS several times with different preps, including full scans (data presented). Our enzyme is active, but no iron signal is detected. Moreover, the experimentally determined structure does not support the presence of a non-heme iron-binding site (Bugg TDH, Ramaswamy S., doi:10.1016/j.cbpa.2007.12.007, and other papers). More detailed response in the recommendations to authors.

(2) Given the metal content reported here, it is important to be able to compare the specific activity of the enzyme reported here with earlier preparations. The authors have now done this in the revised version.

(3) The consumption of formaldehyde to form methylene-THF is potentially interesting, but the authors say "HCHO levels decreased in the presence of THF", which could potentially be due to enzyme inhibition by THF. Is there evidence that this is a time-dependent and protein-dependent reaction? Not yet addressed.

We thank the reviewer for this important point. At present, we have not performed detailed time-dependent or protein-dependent analyses to determine whether the observed decrease in HCHO levels in the presence of THF reflects enhanced downstream consumption or indirect effects, such as inhibition of TDM activity by THF. We acknowledge that further kinetic and protein-dependence studies will be important directions for future work.

Also in Figure 1C, HCHO reduction (%) is not very helpful, because we don't know what concentration of formaldehyde is formed under these conditions; it would be better to quote in units of concentration, rather than %. This point has been addressed by the authors in the revised version.

(4) Has this particular TMAO demethylase been reported before? It's not clear which Paracoccus strain the enzyme is from; the Experimental Section just says "Paracoccus sp.", which is not very precise. There has been published work on the Paracoccus PS1 enzyme, is that the strain used? Details about the strain are needed, and the accession for the protein sequence. Addressed in the revised version.

**Recommendations for the authors:**

**Reviewer #2 (Recommendations for the authors):**

As noted above, there is still a major problem with the proposed mechanism not being feasible, and remaining questions about the presence or absence of a redox-active metal ion in their enzyme. They should:

(1) Re-examine for other metal ions (apart from Zn and Fe) using ICPMS. The redox metal ion could, in theory, be some other transition metal ion.

(2) Seek evidence for the role of metal ions in the activity of this enzyme, for example, by treating with EDTA to remove metal ions, and then adding different metal ions, to correlate activity with a particular metal ion.

We thank the reviewer for these valuable suggestions regarding the metal identity and its functional role in TDM activity. We also acknowledge the reviewer’s concerns regarding the catalytic mechanism. In response, we have substantially revised Scheme 1 and the associated Discussion text to focus on the observed interactions of the substrate (TMAO) and products (DMA and HCHO) within the Zn^2+^-containing active site, rather than proposing a detailed catalytic mechanism that is not fully supported by the current data.

(1) We repeated the ICP–MS analysis using a wide-range full-scan survey to examine the presence of additional metal-associated isotopes beyond Zn and Fe. The corresponding experimental details have been added to the revised ICP–MS Methods section (Page 9). Full-scan ICP–MS profiling of purified TDM detected Zn as the predominant associated metal species (Author response image 1A). In contrast, signals corresponding to Fe and other transition metals were either undetectable or present only at trace levels comparable to, or lower than, those observed in the digested HNO_3_ solution control. To further validate this observation, we performed targeted ICP–MS quantification for both Zn and Fe on the same purified samples. These measurements confirmed that Fe was below the detection threshold, whereas Zn was consistently detected at an approximate ratio of 0.5 Zn^2+^ per protein monomer (Author response image 1B, C, Figure S5).

The observed 0.5 Zn^2+^-to-protein stoichiometry is consistent with the previously discussed 2 full-length + 2 half-domain (2+2½) assembly. In this complex, only the intact core domains retain the complete metal-binding motif, whereas the truncated half-domains lack the Zn^2+^-binding region. Consequently, only two metal-binding sites are expected per assembled complex, in agreement with the ICP–MS measurements. We additionally note that Zn^2+^ was not intentionally supplemented during purification. Based on the current cryo-EM and biochemical data, both metal-binding sites in the full-length subunits appear similarly occupied, with no evidence for asymmetric metal loading.

Importantly, the previously published FEBS Journal model proposed an Fe^2+^-binding site based on metal analysis and homology modeling rather than direct experimental determination. The residues implicated in Fe^2+^ binding are well resolved in our experimental maps and do not define a metal-coordination environment compatible with a second mononuclear metal-binding site. Consistent with the ICP–MS results, the experimental structure provides no evidence of a second metal-binding site. Nevertheless, the enzyme remains catalytically active under these conditions.

(2) We attempted metal depletion experiments using EDTA treatment to evaluate the functional role of the bound metal ion. However, removal of metal ions resulted in rapid protein aggregation, preventing subsequent activity measurements. These observations suggest that the bound Zn^2+^ ion plays an important role in maintaining the structural integrity and stability of the TDM complex. While metal reconstitution experiments would be informative, the aggregation observed following metal depletion precluded a meaningful assessment of alternative metal ions in the current study.

**Author response image 1.**
